# Application of large language models as decision support tools in occupational health and safety management: a cohort study of industrial workers

**DOI:** 10.3389/fpubh.2026.1815316

**Published:** 2026-05-08

**Authors:** Francesca Vella, Paola Senia, Veronica Filetti, Ermanno Vitale, Giuseppe Sferrazzo, Lorenzo Privitera, Venerando Rapisarda, Giuliano Indelicato, Nektaria Zagorianakou, Stefania Durante, Lucia Rapisarda

**Affiliations:** 1 Department of Medicine and Surgery, Occupational Medicine, University of Enna Kore, Enna, Italy; 2 Occupational Medicine Unit, Department of Clinical and Experimental Medicine, University of Catania, Catania, Italy; 3 Provincial Health Authority of Catania, Catania, Italy; 4 Department of Nursing, School of Health Sciences University of Ioannina, Ioannina, Greece; 5 Anesthesia and Resuscitation Unit, Cannizzaro Hospital, Catania, Italy

**Keywords:** artificial intelligence, fitness for work, large language models, occupational health, risk assessment

## Abstract

**Background:**

Occupational health and safety (OHS) risk assessment is a core preventive process aimed at identifying workplace hazards, estimating risks, and implementing control measures to reduce occupational injuries and diseases. Recent evidence indicates that AI-based systems may assist hazard identification, risk prioritization, and preventive planning, improving efficiency and standardization.

**Objectives:**

This study compared AI outputs with occupational physician (OP) analyses in risk assessment, health surveillance protocol drafting, and fitness-for-work determinations.

**Methods:**

This retrospective observational study was conducted in a multinational construction and facility management company with approximately 200 employees. An LLM-based system was evaluated for occupational risk assessment, health surveillance protocol development, and fitness-for-work decisions through structured comparison with an experienced OP. Three objectives were addressed: (1) analysis of the company risk assessment document (RAD); (2) comparison of surveillance protocols for specific tasks; (3) quantitative assessment of agreement in fitness-for-work judgments. The AI system (Perplexity Pro^®^, “Deep Research”) was used. Agreement was measured using Cohen’s Kappa.

**Results:**

AI-generated and OP-generated risk assessments were fully concordant (100%). Risk distribution across job categories was consistent, with high overall concordance (93%). Differences in surveillance protocols reflected regulatory interpretation and contextual exposure assessment rather than omission of clinically relevant elements.

**Conclusion:**

LLM-based AI can reliably support standardized occupational health decisions when applied to structured data. Despite high concordance in risk assessment, protocol development, and fitness-for-work judgments, regulatory interpretation and contextual clinical evaluation remain dependent on human expertise. AI should therefore be considered a complementary decision-support tool in occupational health practice.

## 1 Introduction

Occupational health and safety (OHS) risk assessment represents a fundamental preventive process aimed at identifying workplace hazards, estimating associated risks, and implementing appropriate control measures to reduce occupational injuries and work-related diseases (1, 2). A well-structured risk assessment is widely recognized as a prerequisite for effective prevention strategies and regulatory compliance, forming the basis for health surveillance programs and organizational safety policies (3, 4). Despite its central role, multiple studies have documented substantial variability in the quality, completeness, and methodological rigor of risk assessment practices across different sectors and organizational contexts, potentially limiting their effectiveness in injury and disease prevention (5–7).

International and European Institutional Bodies have repeatedly emphasized the need for standardized, practical, and evidence-based approaches to occupational risk assessment, particularly to support employers operating in complex, high-risk, or rapidly evolving work environments (8, 9).

Digital transformation has been identified as a key enabler for improving consistency, traceability, and updating of risk assessment processes, especially in small and medium-sized enterprises where resources and specialist expertise may be limited (10). In parallel, occupational risk management research has progressively shifted toward structured, data-informed models designed to improve decision-making reliability and transparency (11, 12).

Within this evolving landscape, artificial intelligence (AI) has emerged as a promising tool capable of enhancing OHS practices through automated data processing, pattern recognition, and scalable analytics applied to large volumes of safety-related information (13, 14). Recent evidence suggests that AI-based systems may support hazard identification, risk prioritization, and preventive planning, offering potential benefits in terms of efficiency and standardization (15, 16).

However, systematic reviews indicate that real-world evidence regarding the impact of AI-assisted OHS tools on worker health outcomes remains limited, heterogeneous, and often context-dependent (17–19).

Advances in generative AI, particularly large language models (LLMs), have further expanded the feasibility of automated analysis of unstructured textual data, enabling the extraction, synthesis, and interpretation of complex technical, regulatory, and clinical documentation (20–22). In healthcare settings, LLMs have shown promising performance as clinical decision support tools, supporting diagnostic reasoning, documentation review, and risk stratification when appropriately supervised by human experts (23–26). Nevertheless, the deployment of generative AI in occupational health contexts raises critical concerns related to transparency, explain ability, data protection, algorithmic bias, and medico-legal accountability, particularly when AI-generated outputs may influence fitness-for-work judgments or health surveillance decisions (27–30).

From an occupational medicine perspective, the assessment of fitness for work remains a complex medico-legal act that integrates clinical findings, exposure assessment, regulatory requirements, and professional judgment by the occupational physician (OP) (31, 32). While AI systems can process large datasets with speed and consistency, they currently lack contextual awareness and experiential reasoning, underscoring the importance of evaluating AI as a complementary decision support tool rather than a replacement for professional expertise (33, 34).

Accordingly, rigorous empirical studies comparing AI-generated outputs with expert OP judgments are required to clarify the appropriate scope, limitations, and added value of AI-assisted decision-making in real-world occupational settings (35–37). The aim of this study was to compare AI with the occupational physician analyses for risk assessment, the drafting of health surveillance protocols and the definition of suitability for the specific job.

## 2 Methods

### 2.1 Study design

This retrospective observational study was conducted within a multinational company operating in the construction and facility management sector, with a focus on an Italian operational site specialized in the design, construction, and maintenance of electrical networks. The workforce consisted of approximately 200 employees distributed across administrative, technical, and operational roles. Formal authorization for AI-based document analysis was obtained from the company prior to data processing, and all documents were anonymized and converted into PDF format.

Ethical committee approval was not required, as occupational risk assessment and health surveillance activities are regulated by national occupational safety legislation (Legislative Decree 81/2008). All processed data were fully anonymized prior to analysis, and informed consent was obtained from each participating worker.

**Figure 1:**
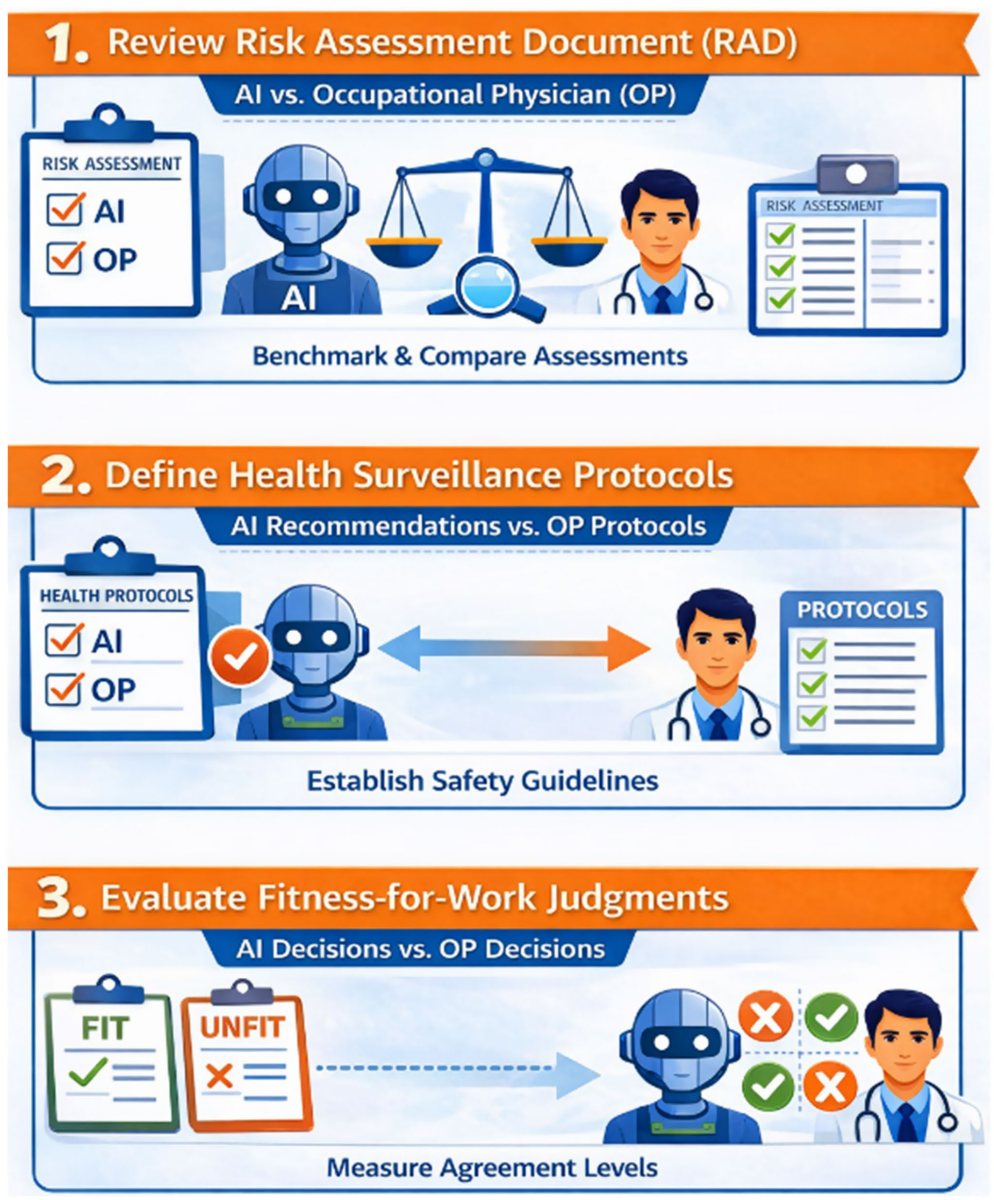
Workflow comparing artificial intelligence (AI) and Occupational Physician (OP) outputs: 1. risk assessment evaluation; 2. definition of health surveillance protocols; 3. evaluation of fitness-for-work judgments.

In this context, the study evaluated the use of an LLM–based system for occupational risk assessment, health surveillance protocol development, and fitness-for-work decision-making, through a direct and structured comparison with the outputs of an experienced OP. The study had three specific objectives (see Figure 1): (1) to critically examine an existing company occupational risk assessment document (RAD) by benchmarking AI-generated assessments against those produced by the OP; (2) to establish health surveillance protocols for each task to specific occupational risks by comparing AI-based recommendations with physician-defined protocols; (3) to quantitatively assess the level of agreement between fitness-for-work judgments for specific job tasks issued by the OP and those generated by the AI.

### 2.2 Artificial intelligence application for risk assessment document analysis -objective 1

The AI system used in this study was Perplexity Pro^®^, employing the “Deep Research” module for advanced document intelligence. The platform integrates natural language processing capabilities to analyze complex textual and tabular content, including risk matrices and technical diagrams embedded in PDF files. It enables semantic decomposition of risk assessment documentation and identification of relationships among regulatory provisions, risk classifications, and preventive measures. A three-level validation process was implemented, consisting of verification of the current validity of cited legal provisions, comparison with the most recent national occupational safety guidelines, and consistency analysis with relevant jurisprudence. Data protection measures included AES-256 encryption, automatic anonymization of identifying information, and guaranteed deletion of uploaded documents within 72 h.

The existing RAD was analyzed by the AI system using a structured framework aligned with national occupational safety legislation. The analysis was based on predefined 12 questions addressing document identification and purpose, completeness of mandatory elements, legal compliance, classification of occupational risks, application of the hierarchy of controls, evaluation of residual risk, internal coherence, and identification of missing or underestimated hazards. This approach aimed to assess the ability of the AI system to support occupational safety professionals in the improvement of risk assessment documentation.

### 2.3 Health surveillance protocols and fitness-for-work judgments-objective 2

For each identified job category, the health surveillance protocols proposed by the AI system were compared with those defined by the OP, focusing on the type and frequency of clinical examinations, instrumental tests, and screening procedures in relation to specific occupational risks. The OP had more than 20 years of professional experience and had been responsible for the company’s occupational health surveillance activities for over 3 years. Although the final medico-legal responsibility remained assigned to the designated occupational physician (Legislative Decree 81/2008), the evaluation process was supported by collegial clinical discussion with two additional occupational physicians (P. S. and F. V.), each with over 10 years of professional experience.

### 2.4 Study population and OP assessment-objective 3

The study population included workers employed at the company for at least 3 years. Inclusion criteria were current employment at the company, a minimum employment duration of 3 years, and provision of informed consent for data processing. Exclusion criteria included shorter employment duration or absence of informed consent. Anonymized occupational health records and risk-related documentation were independently analyzed by both the AI system and an experienced OP.

Health surveillance was conducted in accordance with national occupational health legislation and included workplace inspections, clinical examinations, assessment of exposure-related risks, and formulation of fitness-for-work judgments. Site inspections were performed both in administrative facilities and in temporary and mobile construction sites.

Fitness-for-work judgments were classified into four categories: fit for work, fit for work with limitations, fit for work with prescriptions and un fit. Judgments generated by the AI system were compared with those independently formulated by the OP.

### 2.5 Statistical analysis

Descriptive statistics were used to summarize demographic, clinical, and occupational characteristics of the study population. Continuous variables were expressed as mean ± standard deviation. Agreement between AI-generated and physician-generated fitness-for-work judgments was assessed using Cohen’s Kappa (*κ*) statistic. The standard error of κ and the corresponding 95% confidence interval were calculated to evaluate the precision and statistical significance of the observed agreement. Interpretation of κ values followed the Landis and Koch classification, ranging from poor agreement to almost perfect agreement.

## 3 Results

### 3.1 Objective 1

Occupational risk assessment performed autonomously by the AI system and the analysis of the pre-existing RAD conducted by both the AI system and the OP yielded fully concordant (100%) results. Across all occupational categories, the following risk domains were systematically identified: mechanical and electrical hazards; tripping, impact, slipping, and limited accessibility; falls from height; traffic- and vehicle-related risks; uncontrolled moving parts and crushing hazards; fire and explosion hazards; dust exposure; noise; night and shift work; inadequate lighting; hand–arm and whole-body vibration; chemical agents; physical agents; hazardous substances; biological risk; video display terminal exposure; pregnancy-related risks; manual handling of loads; and psychosocial risk factors (see Table 1).

**Table 1:** RAD quality strengths and limitations assessed by AI using a predefined 12-item checklist.

Item	Checklist domain	Key AI findings	Rating
1	Document type, context, and purpose	Correctly identified as a mandatory occupational risk assessment document; purpose and scope described.	C
2	Drafting date and update/revision status	Drafting date present; no documented updated version/date; revision mentioned only in general terms.	PC
3	Company identification data	Company identifiers (business details, activity code, workforce, employer) clearly reported.	C
4	Summary of mandatory RAD contents	Main hazards by job category and preventive/protective measures reported; safety roles and training described.	C
5	Strengths vs. missing/inconsistent elements	Strengths: clear structure, hazards mapped, roles/measures reported. Gaps: no planned periodic updating; worker consultation not documented; incomplete biological/WBV assessment.	PC
6	Sector/legal compliance	References to national legislation/guidelines were appropriate, but some measures were mentioned without practical implementation (e.g., psychosocial/organizational measures).	PC
7	Risk classification	Risks classified by job and category; however, missing quantitative noise/vibration data and limited detail for biological/WBV.	PC
8	Language clarity and orthographic quality	Technical language generally appropriate, but repetitions/typos and limited operational readability; lacking practical summaries/schematics.	PC
9	Gaps and omissions	Missing instrumental measurements (noise/vibration), incomplete chemical characterization (e.g., exposure details), limited biological/EMF depth; psychosocial tools and worker consultation/update documentation not adequately reported.	NC
10	Hierarchy of controls (collective vs. PPE)	Frequent preference for PPE over collective controls; insufficient emphasis on engineering/collective controls (e.g., ventilation systems).	NC
11	Residual risk appraisal	Residual risk addressed qualitatively, without quantified effectiveness of controls; residual risk remained potentially high for several hazards.	NC
12	Potentially neglected risks	Under-addressed or insufficiently developed risks included biological exposures, whole-body vibration, psychosocial risks, EMF, and other context-dependent hazards.	NC

C, Compliant; PC, partially compliant; NC, not compliant.

Using the predefined 12-item assessment checklist, the existing RAD showed adequate compliance for document identification and basic administrative content (items 1, 3, and 4). However, several deficiencies emerged in elements relevant to document governance, exposure characterization, and the operationalization of risk control measures. Specifically, the RAD lacked a clearly documented plan for periodic review and did not fully document worker consultation (items 2 and 5). Quantitative exposure data for noise and vibration were absent, and the coverage of selected hazards - particularly biological risk and whole-body vibration-was incomplete (items 7 and 9). The analysis further identified no planned periodic updating; worker consultation not documented (item 5) and limited implementation details for psychosocial risk management (items 6, 9, and 12). Finally, the RAD frequently prioritized personal protective equipment over collective control measures, provided only qualitative residual-risk appraisal, and showed inconsistencies between hazard identification and the associated control strategies (items 10 and 11).

### 3.2 Objective 2

The distribution of risk factors across job categories was consistent between AI-based and physician-based assessments, with no discrepancies in the identification or categorization of occupational hazards. Based on task analysis and exposure profiles, workers were classified into eight occupational categories: electrician, construction worker, heavy vehicle driver, warehouse worker, mechanic, administrative employee, area manager/line supervisor, and technical manager (see Table 2).

**Table 2:** Occupational risks across job roles assessed by OP and AI, with agreement analysis.

Job role	AI risk assessment	OP risk assessment	Concordance	Differences*
Electrician	ME, Tis, Fh, Uc, Fe, ER, De, N, HAV, Ca, Br, MHL, Psf, HS	ME, Tis, Fh, Uc, Fe, ER, De, N, HAV, Ca, Br, MHL, Psf, HS, Pa	93%	Pa
Construction worker	Me, Tis, Fh, Uc, Fe, ER, De, N, HAV, HS, Ca, Br, MHL, Psf, WBV, NW	Me, Tis, Fh, Uc, Fe, ER, De, N, HAV, HS, Pa, WBV, Ca, Br, MHL, Psf, NW	94%	Pa
Heavy vehicle driver	ME, Tis, Tvle, Uc, Fe, ER, De, HAV, Ca, Br, MHL, Psf, WBV, NW	ME, Tis, Tvle, Uc, Fe, ER, De, HAV, WBV, Ca, Br, MHL, Psf, NW, RpS, Pa	87,5%	RpS, Pa
Warehouse worker	WVT, PBW, ME, Tis, Fh, Tvle, Uc, Fe, ER, De, N, HAV, WBV, Ca, Pa, HS, Br, MHL, Psf, NW, L, N	WVT, PBW, ME, Tis, Fh, Tvle, Uc, Fe, ER, De, N, HAV, WBV, Ca, Pa, HS, Br, MHL, Psf, NW, L, N, RpS	86%	RpS
Mechanic	ME, Tis, Uc, Fe, L, ER, De, N, HAV, Ca, Br, MHL, Psf	ME, Tis, Uc, Fe, L, ER, De, N, HAV, Ca, Br, MHL, Psf, Pa	93%	Pa
Administrative employee	Tis, Tvle, Uc, Fe, L, Psf, WVT, PBW	Tis, Tvle, Uc, Fe, L, Psf, WVT, PBW	100%	–
Area manager/line supervisor	Tis, Tvle, Uc, Fe, L, WBV, Psf, WVT, PBW	Tis, Tvle, Uc, Fe, L, WBV, Psf, WVT, PBW	100%	–
Technical manager	Tis, Tvle, Uc, Fe, L, De, N, WBV, Ca, Br, Psf, WVT	Tis, Tvle, Uc, Fe, L, De, N, WBV, Pa, Ca, Br, Psf, WVT	92%	Pa

*Further risks identified by the OP; Br, Biological risk; Ca, Chemical agents; De, Dust exposure; ER, Ergonomic risk—inappropriate postures and/or biomechanical overload; Fe, Fire and explosion hazards; Fh, Falls from height; HAV, Hand–arm vibration; HS, Hazardous substances; L, Lighting; ME, Mechanical and electrical hazards; MHL, Manual handling of loads; N, Noise; NW, Night work and shift work; Pa, Physical agents; PBW, Pregnancy and breastfeeding-related risks; Psf, Psychological factors; RpS, Third party safety liability risk; Tis, Trips, impacts, slips, and difficult access; Tvle, Traffic, vehicles, and lifting operations; Uc, Uncontrolled moving parts and crushing hazards; WBV, Whole-body vibration; WVT, Work with display screen equipment (DSE work).

A comparison of the risks identified for each job role by the AI and the OP shows a high average concordance (93%). Some differences were noted in the risk assessment for each job role. In particular, the OP detected the presence of risk from exposure to physical agents (Pa) for the roles of electrician, construction worker, heavy vehicle driver, mechanic, and technical manager, specifically climatic agents and microclimate-related agents in general. However, for the roles of heavy vehicle driver and warehouse worker, the OP detected the presence of third-party safety liability risk (RpS). This risk is present in all activities involving the operation of mechanical vehicles. This risk was not detected by the AI.

Comparison between health surveillance protocols generated by the AI system and those defined by the OP demonstrated partial divergence depending on occupational role (see Table 3).

**Table 3:** Health surveillance protocols (HSP) by job role: AI-generated vs.OP-defined protocols.

Job role	AI-generated HSP	OP-defined HSP	Differences
Electrician	OHME, AUD, SPI, MSE, EMC	OHME, AUD, SPI, MSE, EMC, ALC, TET-IgG	ALC and TET-IgG tetanus (OP)
Construction worker	OHME, AUD, SPI, MSE, EMC, ECG	OHME, AUD, SPI, MSE, EMC, ALC, ECG, TET-IgG	ALC and TET-IgG tetanus (OP)
Heavy vehicle driver	OHME, AUD, MSE, EMC, ECG	OHME, AUD, MSE, EMC, ECG, DRUG, TET-IgG	DRUG (OP)
Warehouse worker	OHME, AUD, MSE, EMC, ECG	OHME, AUD, MSE, EMC, ECG, DRUG	DRUG (OP)
Mechanic	OHME, AUD, SPI, ECG, EMC, MSE	OHME, AUD, SPI, ECG, EMC, MSE, TET-IgG	TET-IgG tetanus (OP)
Administrative employee	OHME, VDT	OHME, VDT	None
Area manager/line supervisor	OHME, VDT, WRS	OHME, VDT, WRS	None
Technical manager	OHME, AUD, EMC, MSE, VDT, ECG	OHME, AUD, EMC, MSE, VDT, ECG, TET-IgG	TET-IgG tetanus (OP)

ALC, alcohol use screening; AI, Artificial Intelligence; AUD, Audiological assessment (otoscopy + audiometry); DRUG, Drug screening (substances of abuse); ECG, Electrocardiogram; EMC, Routine blood tests (hematology/biochemistry); HSP, Health Surveillance Protocol; MSE, Musculoskeletal/spine evaluation; OHME, Occupational health medical examination; OP, Occupational Physician; SPI, Spirometry; TET-IgG, Anti-tetanus IgG serology (tetanus immunity assessment); VDT, Vision assessment for display screen equipment (DSE) work (VDT); WRS, Work-related stress assessment.

The OP included alcohol screening due to work performed at heights above 2 m and assessment of tetanus immunization status for multiple operational roles, in accordance with national regulatory requirements and on-site exposure evaluation. The OP also introduced the requirement for tetanus screening (TET-IgG) for mechanics and technical managers. For heavy vehicle drivers and warehouse workers, the OP also introduced the requirement for screening for narcotic and psychotropic substance use (DRUG). No discrepancies were observed for administrative and managerial roles, for which identical surveillance protocols were proposed by both evaluators. Overall, differences primarily reflected regulatory interpretation and contextual exposure assessment rather than omission of clinically relevant evaluations.

### 3.3 Objective 3

**Figure 2:**
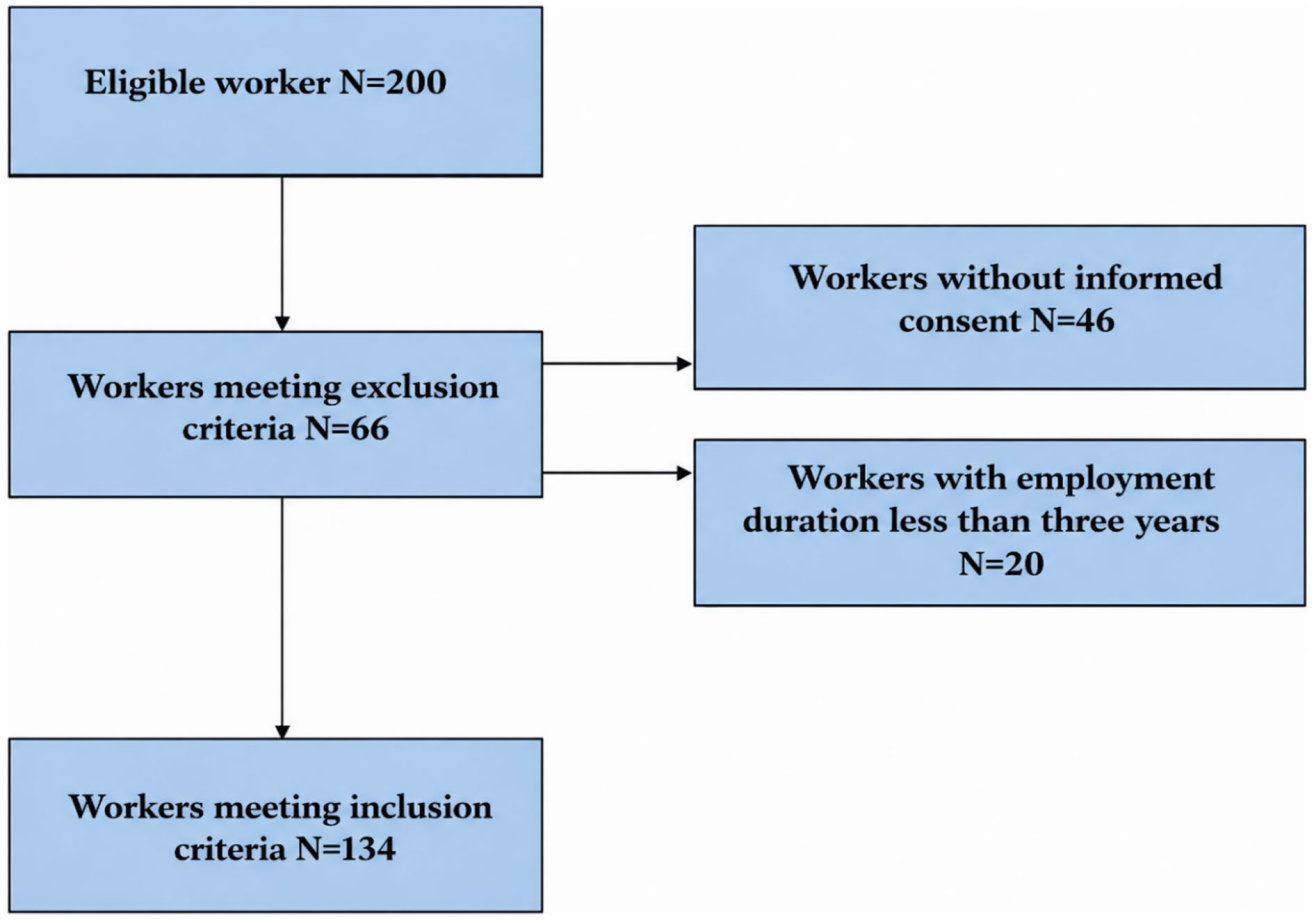
Flowchart of study population according to exclusion and inclusion criteria.

On a total of 200 eligible workers (100%), 134 subjects (67%) met the inclusion criteria and were enrolled in the study. Sixty-six workers (33%) were excluded due to lack of informed consent (*n* = 46; 70%) or employment duration shorter than 3 years (*n* = 20; 30%). Figure 2 shows the flowchart of sample recruitment.

The study population consisted exclusively of male workers, with a mean age of 46.3 ± 9.9 years (range: 22–67 years) and a mean employment seniority of 13.4 ± 3.6 years. Current smokers accounted for 40% (*n* = 54) of the sample. The mean body mass index (BMI) was 28.9 ± 3.1 kg/m^2^. At least one or more chronic medical condition was documented in 58% of workers (*n* = 78). As shown in Table 4, the most prevalent conditions were musculoskeletal disorders involving the spine and/or limbs (35%), obesity (27%), hypertension (21%), diabetes mellitus (6%), myocardial infarction sequelae (6%), renal lithiasis (4%), neoplastic disease (3%), and neurodegenerative disorders (2%). The sample was distributed across the eight occupational categories, as follows: electrician (*n* = 43), construction worker (*n* = 28), heavy vehicle driver (*n* = 32), warehouse worker (*n* = 1), mechanic (*n* = 20), administrative employee (*n* = 2), area manager/line supervisor (*n* = 3), and technical manager (*n* = 5).

**Table 4:** Characteristics of study population.

Characteristics	Subjects *n* = 134
Mean age (years)	46.3 ± 9.9
Sex (male)	134 (100%)
Seniority work (years)	13.4 ± 3.6
Current smokers	54 (40%)
Former smokers	23 (17%)
BMI (cm^2^/kg)	28.9 ± 3.1
Workers with chronic medical condition	78 (58%)
Spine and/or limbs disease	27 (35%)
Obesity	21 (27%)
Hypertension	16 (21%)
Diabetes mellitus Myocardial infarction sequelae Renal lithiasis Neoplastic disease Neurodegenerative disorders	4 (6%) 4 (6%) 3 (4%) 2 (3%) 1 (2%)

Fitness for specific tasks, issued by the OP following health surveillance, were divided as follows: fit for work in 72% (*n* = 96) of cases, fit for work with limitations in 20% (*n* = 27), and fit for work with prescriptions in 8% (*n* = 11). The AI system independently produced an identical distribution of judgments across all categories (see Table 5).

**Table 5:** Fitness for specific tasks: AI vs.OP.

	OP: fit for work	OP: limitations	OP: prescriptions	Total
AI: fit for work	96	0	0	96
AI: limitations	0	27	0	27
AI: prescriptions	0	0	11	11
Total	96	27	11	134

**Figure 3:**
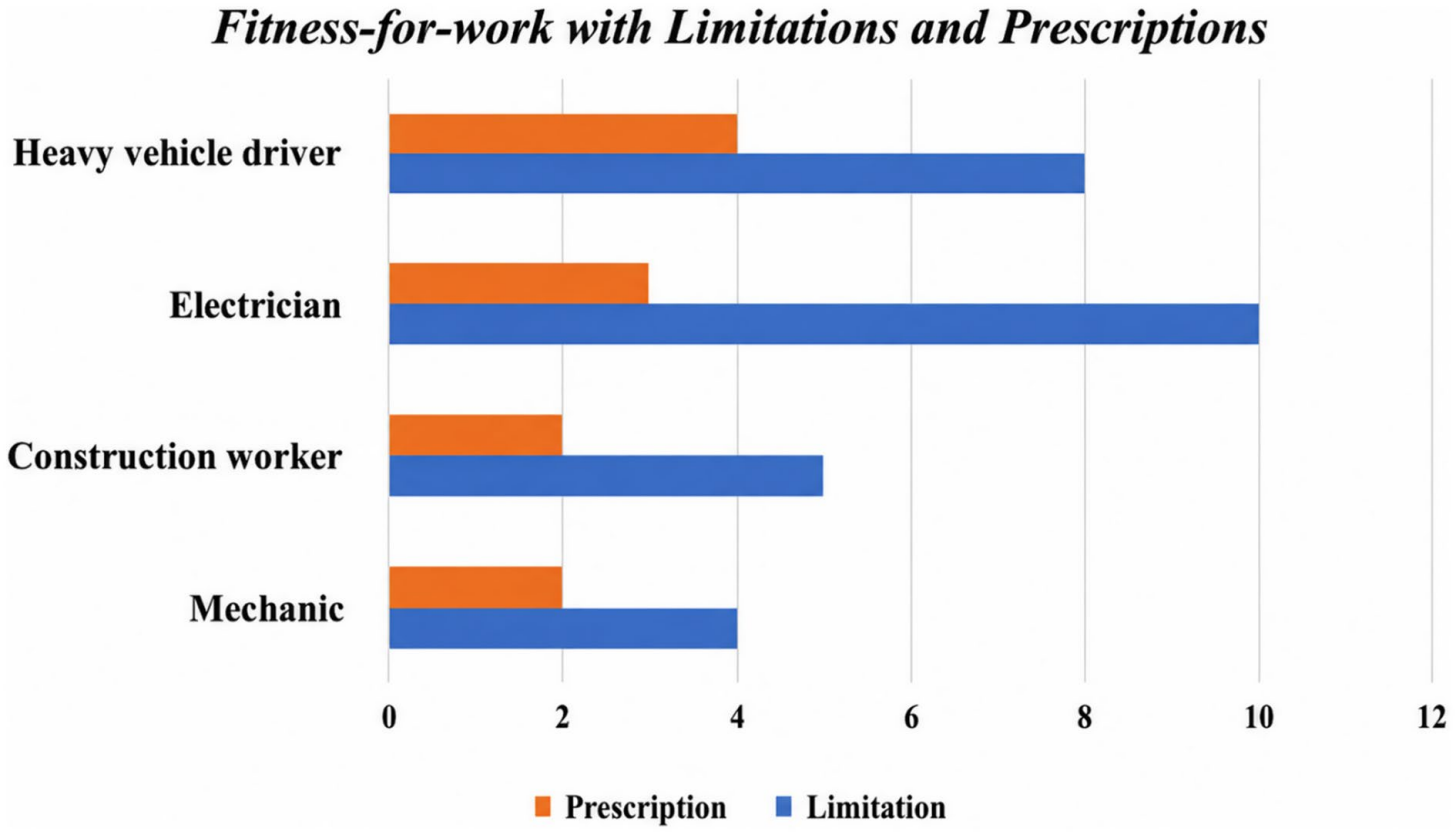
Fitness-for-work with Limitations and Prescriptions distributed across different job role.

Figure 3 shown how fitness-for-work limitations and prescriptions are distributed across the different job roles within the study population. Limitations identified by both evaluators included restrictions on manual load handling (10%; *n* = 14), contraindication to work at heights exceeding 2 m (6%; *n* = 9), and limitations related to shift work (2%; *n* = 3). Prescriptions were exclusively related to shortened intervals for health surveillance follow-up (<12 months) in 8% (*n* = 11) of workers.

The comparison between AI-generated and physician-generated fitness-for-work judgments demonstrated complete concordance across the entire study population. The Cohen’s Kappa coefficient was equal to 1.00, indicating perfect inter-rater agreement.

## 4 Discussion

AI in occupational health is transforming how organizations protect workers, prevent injuries, and ensure compliance (38). Although the integration of AI in the field of occupational health and safety is still in its infancy, it finds numerous applications in the workplace. Some of these applications offer numerous benefits for workers’ health and safety, such as continuous monitoring of workers’ health and safety and the working environment through wearable devices and sensors (39). With the increasing prevalence of AI systems, employers managing employee health and wellness are eager to implement AI to improve efficiency, streamline operations, protect workers, and provide greater resources to healthcare providers in the industry. However, the use of AI is not without risks and requires careful evaluation and diligence before implementation (40). The rapid integration of AI into occupational health and safety represents a paradigm shift in how risk assessment, health surveillance, and medico-legal decision-making may be supported in complex industrial settings. In this context, the present study provides real-world evidence on the performance of an LLM–based system when applied to occupational risk management and fitness-for-work evaluations (21, 24, 25).

Our findings demonstrate a complete concordance between AI-generated and occupational physician–generated fitness-for-work judgments, with a Cohen’s Kappa coefficient of 1.00, indicating perfect agreement. This result aligns with emerging literature suggesting that AI-based decision support systems can replicate standardized clinical reasoning processes when applied to structured datasets and well-defined decision rules (21, 24, 25). Previous studies in clinical and occupational health contexts have shown that machine learning and AI systems perform best in scenarios characterized by high procedural standardization and clearly defined outcome categories, such as risk stratification or eligibility assessments (23, 35). In the present study, the AI system demonstrated full consistency with the OP not only in overall fitness-for-work classification but also in the attribution of limitations and prescriptions, particularly those related to manual handling of loads, work at height, and work shifts. These findings are consistent with evidence suggesting that AI systems can effectively support rule-based and guideline-driven decisions in occupational medicine (13, 14). Recently, several studies have shown the possibility of improving ergonomic analysis through the combined use of AI and wearable sensors (41–44). Similar levels of agreement have been reported in healthcare settings where AI tools were used as clinical decision support systems under expert supervision (34). Conversely, discrepancies observed in the definition of health surveillance protocols—specifically regarding toxicological screening and tetanus immunization—highlight limitations already described in the literature concerning AI performance in regulatory interpretation and context-dependent decision-making (17, 18, 32). Occupational health regulations often require integration of legal texts, interpretative agreements, and on-site exposure assessment, elements that remain challenging for AI systems lacking direct environmental observation and experiential judgment (28). This confirms previous concerns regarding the “black box” nature of AI and its limited ability to autonomously manage medico-legal responsibilities (27). The AI-based analysis of the RAD further corroborated existing evidence on the usefulness of document intelligence systems in identifying structural weaknesses, missing elements, and inconsistencies in safety documentation (11, 37). The ability of the AI system to detect underestimation of biological, vibration, and psychosocial risks mirrors findings from recent occupational safety studies emphasizing the role of digital tools in enhancing RAD quality and regulatory compliance (9). A major strength of this study lies in its real-world application within a complex industrial setting, involving a heterogeneous workforce with a high prevalence of chronic conditions. Unlike simulation-based or theoretical evaluations, this investigation directly compared AI outputs with those of an experienced OP operating under routine professional conditions (13). The use of fully anonymized clinical and risk documentation, combined with a structured statistical assessment of agreement, enhances the methodological robustness of the findings. Furthermore, the study addresses an underexplored area of occupational medicine, namely the evaluation of AI systems in fitness-for-work judgments, which represent a high-stakes medico-legal act (39). AI systems are already used in reading X-rays, CT scans, ECGs and other tests to detect occupational pathologies, such as musculoskeletal injuries or pneumoconiosis, early (45, 46). Several limitations should be acknowledged. First, the study was conducted in a single company within a specific industrial sector, potentially limiting generalizability to other occupational contexts. Second, all workers were male, reflecting workforce composition but restricting applicability to mixed-gender populations (40, 47–49).

Although the evaluation process was supported by collegial discussion with additional experienced occupational physicians, partially mitigating individual evaluator bias, formal inter-rater reliability metrics involving multiple independent raters (e.g., multi-rater Cohen’s kappa or ICC) were not calculated.

Finally, the AI system relied exclusively on document-based inputs and could not incorporate direct workplace inspections or dynamic clinical assessments, which remain central to OP practice.

## 5 Conclusion

In conclusion, this study provides novel empirical evidence that LLM-based AI systems can achieve complete concordance with expert OP in fitness-for-work judgments when applied to structured occupational health data. While AI demonstrated substantial potential as a decision support tool for RAD analysis and standardized medical judgments, it remains insufficient for autonomous regulatory interpretation and context-dependent clinical decision-making. However, in order to guarantee responsible and equitable deployment, their incorporation into healthcare systems also presents difficult conundrums that need to be carefully handled.

These findings reinforce the role of AI as a complementary - rather than substitutive - tool in occupational health practice, while preserving the central role of human judgment in safeguarding workers’ health and safety.

Future research should focus on multicenter studies, integration of real-time exposure data, and development of explainable AI models capable of supporting, rather than replacing, professional responsibility in occupational medicine.

By rigorously evaluating concordance, discrepancies, and decision-making patterns, this study aims to generate robust empirical evidence on the capabilities and limitations of LLM-based systems as clinical decision support tools in occupational health.

The future will not see AI replace the occupational physician, but rather the occupational physician will have to be able to use AI, with a critical and controlling spirit, as a further step forward for the health and safety of workers.

## Data Availability

The raw data supporting the conclusions of this article will be made available by the authors, without undue reservation.
